# Supplementary material for: Next-Generation Sequencing for Cystic Fibrosis: Florida Newborn Screening Experience
Source: Int J Neonatal Screen. 2025 Oct 14;11(4):94. doi: 10.3390/ijns11040094 (PMC12551084; doi:10.3390/ijns11040094)
Supplement: Supplementary file 1 [file IJNS-11-00094-s001.zip › IJNS-3761101-supplementary.pdf]

Newborn Screening for Cystic Fibrosis (CF) Single Variant Results

HOW DOES FLORIDA NEWBORN SCREENING PROGRAM (NBS) SCREEN FOR CF?

CF screening involves up to 3 tiers of testing before reporting results.

First Tier Screening

Every specimen is tested using immunoreactive trypsinogen (IRT). If the IRT results are < 50 ng/ml, CF will report as “Within Normal Limits– DNA Not Performed” on the newborn screening report.

Second Tier Screening

DNA analysis of the 72 most common CF-causing variants is performed for the following reasons:

- Top 4% of IRT results each day,
- All specimens with IRT results ≥ 50 ng/ml , and
- All specimens with a meconium ileus reported.

Third Tier Screening

Next Generation Sequencing is performed for the following reasons:

- A CF-causing variant was detected during second tier analysis.
- No variant was detected during second tier analysis, and the IRT level was ≥ 160 ng/ml.
- Second tier results were inconclusive.

WHAT IS A REPORTABLE VARIANT?

A reportable variant is classified as either pathogenic, likely pathogenic, or a variant of uncertain significance.

If only a **single reportable variant** is detected after all three tiers of testing, the **infant is considered to be a CF carrier**. Letters are mailed to the parent/guardian and primary care provider (PCP) on record to advise of the results.

If **two reportable variants** are detected, the infant will be referred by the NBS Program to a contracted CF Referral Center for additional testing. The infant will need a sweat test to make or rule out a diagnosis of CF or CF Related Metabolic Syndrome. (See reverse for contact information.)

SPECIAL CONSIDERATIONS FOR SCREENING CF RESULTS.

IRT levels can fluctuate and may not require DNA analysis on all specimens; however, DNA results do not change. Any CF variant results detected on any specimen are valid and should be treated as such.

While Florida’s variant panel can detect known CF-causing variants, it is possible for an infant to have a variant(s) not identified through screening. Physician discretion is advised if signs and symptoms are present, or there is a family history of CF.

Prenatal testing through commercial laboratories is often limited to the most common CF-causing variants, so it is possible for newborn screening to detect a variant not included on prenatal testing panels.

WHAT HAPPENS NEXT?

- Ensure screening results are included in the infant’s medical record.
- Family planning and genetic counseling should be provided, ideally by a licensed genetic counselor.
- There is no further testing required at this time; however, additional testing to confirm screening results may be completed, if desired.
- Sweat testing is the gold standard for CF diagnostic testing and should be completed by a provider accredited by the Cystic Fibrosis Foundation. See the reverse side of this page for a list of contracted NBS CF Referral Centers.
- Assist parents with obtaining their own variant testing, if desired.

DESIRE ADDITIONAL TESTING?

Should additional testing be desired, please contact the contracted CF newborn screening referral center in your area.

NBS CF Referral Center Name, City, and Phone Number:

Johns Hopkins All Children's Hospital St. Petersburg 727-767-4146

Lee Health- Golisano Children's Hospital Ft. Myers 239-437-5500

Memorial Health Joe DiMaggio Children's Hospital Hollywood 954-265-3665

Nemours Children's Health Jacksonville 904-697-3600

Nemours Children's Health Orlando 689-208-5205 (Ask for CF Team.)

Nemours Children's Health Pensacola 850-505-4700

Nicklaus Children's Hospital Miami 305-669-5864

Orlando Health Arnold Palmer Children's Hospital Orlando 321-841-6350

University of Florida Gainesville 352-273-8380

University of Miami Miami 305-243-6162

University of South Florida Tampa 813-821-8029

For additional questions, please call the Newborn Screening Follow-up Program Nursing Unit at **866-804-9166.**

Mission:
To protect, promote and improve the health of all people in Florida through integrated state, county and community efforts.

Florida HEALTH

Ron DeSantis
Governor

Joseph A. Ladapo, MD, PhD
State Surgeon General

Vision: To be the **Healthiest State** in the Nation

Important Information About Your Patient
Letter to PCP CF Carrier

DR. MARY JONES
678 DOWNTOWN AVENUE
ANYTOWN, FL 33333

JANE SMITH
1234 MAIN STREET
ANYTOWN, FL 33333

Report Date:	6/1/2025
Baby's Name:	SMITH, BABY GIRL<br>AKA: SMITH, JESSICA
Birth Date:	5/17/2025 10:00
Lab Specimen #:	250521SF9999
Medical Rec #:	123456789
Parent phone #.	(555) 555-5555

Dear Dr. MARY JONES,

Newborn screening results for this baby showed the presence of a single variant for cystic fibrosis (CF), which is suggestive of being a CF carrier. Genetic counseling with the baby's parent(s) regarding inheritance and family planning should be completed, ideally by a licensed genetic counselor.

Please call the Newborn Screening Follow-up Program at 866-804-9166 with questions or concerns.

You were listed as this baby's primary care provider. If this baby is not your patient, please complete the form below and fax it to 850-922-5385.

Thank you for your assistance.

Sincerely,

Florida Newborn Screening Program
Children's Medical Services

I am not the child's primary care physician. The child's PCP is ________________________________

Telephone number: ________________________________

Fax name of **new** primary care physician to 850-922-5385.
